# The Fhit protein: an opportunity to overcome chemoresistance

**DOI:** 10.18632/aging.101123

**Published:** 2016-11-12

**Authors:** Eugenio Gaudio, Francesco Paduano, Carlo M. Croce, Francesco Trapasso

**Affiliations:** Dipartimento di Medicina Sperimentale e Clinica, University Magna Græcia, Catanzaro, Italy

**Keywords:** Fragile Histidine Triad (FHIT), tumor suppressor, reactive oxygen species, targeted therapy, mimetic peptides

Since the very first moment of its discovery in 1996 [1], the *FHIT (Fragile Histidine Triad)* gene product appeared as a very intriguing molecule to be investigated in the pathogenesis of cancer. In fact, *FHIT* encompasses the most common fragile site in human [2], whose genetic alterations, leading to the loss of *FHIT* expression, have been reported in the majority of human cancers [3]. Other than genetic lesions, *FHIT* expression in both solid and hematopoietic malignancies is also impaired by its promoter hypermethylation [4, 5], making Fhit protein virtually completely lost in tumors. Moreover, several reports have intriguingly pointed to Fhit loss as a very early event in epithelial tumorigenesis (presumably, the earliest event in smoking-related lung cancer) [3].

These findings, along with both chemically induced and spontaneous predisposition to cancer development of Fhit+/− and Fhit−/− mice, respectively [6, 7], have encouraged the application of a gene therapy approach in several experimental models of cancer, both *in vitro* and *in vivo*. We successfully demonstrated that Fhit restoration in cancer cells through recombinant adeno- or adenoassociate viruses was able to trigger apoptosis in a number of cancer types, including esophageal, pancreatic, mammary cancer, and even leukemia, and to block their *in vivo* tumor formation [8–11]. Moreover, Fhit protein was not only curative in Fhit+/− mice bearing NMBA-induced forestomach tumors [12] but it could also prevent tumor formation in Fhit+/− mice treated with the same carcinogen [13, 14]. These results were very interesting as they represented the proof-of-principle that *FHIT* was a therapeutic gene indeed.

Fhit protein function is still partly a mystery; for long time we only knew that it was an enzyme belonging to the HIT (Histidine Triad) protein family, a class of molecules involved in the hydrolysis of dinucleoside three- and tetraphosphate [3]. In order to investigate the role of Fhit hydrolase activity in tumor suppression, we planned an apoptosis quantitative assay based on the design of *FHIT* alleles driven by recombinant adenoviruses. We proved that the Fhit mutants able to bind the substrate but with impaired catalytic activity could still efficiently trigger apoptosis of cancer cells; also, apoptosis was still observed with Fhit mutants unable to bind the substrate, even though to a much lower extent compared to the wild-type protein [15, 16], thus suggesting that Fhit tumor suppression activity in cancer cells could presumably be dependent by different and independent molecular pathways. However, because of the failure to identify protein partners through conventional approaches, no Fhit pathways have ever been described for more than a decade after its discovery. Therefore, this limitation has contributed to lower the interest toward a molecule whose importance in human and experimental tumorigenesis was clearly established.

Finally, in 2008 we published a pivotal study demonstrating for the first time the existence of a Fhit protein complex [17]. We designed an elegant proteomic-based approach in order to identify the Fhit-interacting molecules once Fhit-mediated apoptosis was triggered in cancer cells after adenovirus-mediated *FHIT* restoration. Briefly, a tagged Fhit protein was expressed in lung cancer cells through a recombinant adenovirus; the candidate Fhit protein complex was then stabilized by using DSP, a vital photo-crosslinker able to generate disulfide bonds among the lysines of interacting proteins. Total cell extracts were then purified, digested and used to run a mass-spectrometry analysis. The list of candidate proteins was composed of six proteins only; all of them were tracked in mitochondria, including Hsp10 and Hsp60, also detected in the cytosol. These results described for the first time Fhit mitochondrial subcellular localization through the shuttling of Hsp60 and Hsp10 and, surprisingly, directly put Fhit protein in a pathway involving molecules, such ad ferredoxin reductase (Fdxr), important in the generation of reactive oxygen species (ROS) as a result of the activity of the respiratory chain. This aspect is very important in the physiology of a normal cell, as free radicals represent a protective mechanism toward cell injuries of different nature; in fact, their increase observed during exposure to noxious agents can address normal somatic cells to apoptosis, thus avoiding DNA alterations potentially leading to cancer [18]. These considerations put *FHIT* in a crucial position in an initiated cell, as its loss in the preneoplastic genome predisposes to reduced apoptosis; this allows, in turn, for the accumulation of further genetic lesions because of the reduction in the efficiency of the intrinsic pathway of apoptosis, plausibly due to the reduced extent of free radicals production in Fhit-negative cells [19, 20]. This aspect can also account for the reduced sensitivity to anticancer drugs described in Fhit-negative cancer cells. Thus, *FHIT* reconstitution in malignant cells restores their ability to generate ROS, to trigger apoptosis and, finally, to increase responsiveness to chemotherapy [17].

However, from a therapeutic point of view, the major problem about *FHIT* cancer gene therapy was its full-length reconstitution in tumors through recombinant viruses. In fact, because of its intrinsic limitations (mainly, the inability of the recombinant vectors to target all cancer cells *in vivo*), cancer gene therapy is far from being applicable as a large-scale approach in a clinical setting of solid tumors treatment. This prompted us to search for oncoproteins interacting with Fhit in cancer cells in order to possibly use small parts of its product still able to interfere with the oncogenic pathways driven by them. This aim was pursued by a slight modification of the approach previously used to identify Fhit partners [21]. As Fhit protein is virtually ubiquitously distributed in mammalian cells [17], we searched for novel Fhit partners in cell membranes, paying particular attention to the plasma membrane, the subcellular location where not only the mitogenic signals start but also where chemoresistance can partly find its molecular support [22].

**Figure 1:**
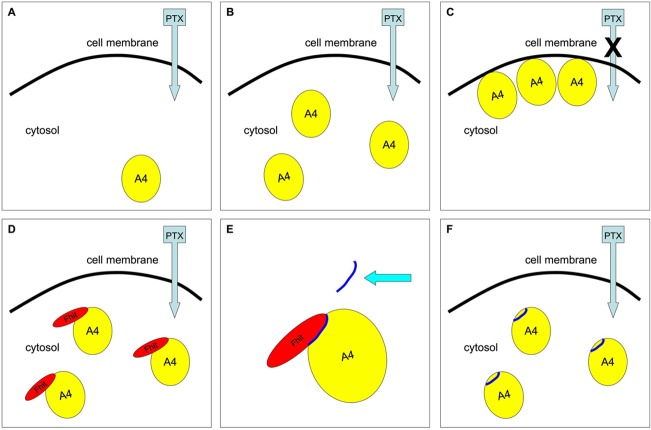
Fhit protein or Fhit-mimetic peptide interaction with ANXA4 restores chemosensitivity to paclitaxel in lung cancer cells This simplistic drawing indicates that ANXA4, preferentially located in the cytosol of Fhit-negative lung cancer cells (**A**), undergoes overexpression (**B**) and translocation (**C**) to the inner side of plasma membrane following to paclitaxel treatment; this effect results in the chemoresistance to the drug (**C**). Fhit overexpression blocks ANXA4 translocation from cytosol to plasma membrane thus restoring sensitivity to paclitaxel (D). The Fhit peptide interacting with ANXA4 (E) recapitulates the effect of the wild-type Fhit protein on chemoresistance both *in vitro* and *in vivo* (**F**).

Interestingly, in the novel list of candidate Fhit partners we found annexin A4 (also known as ANXA4 or A4), a protein belonging to the family of annexins that are molecules able to bind calcium ions and phospholipids [23]. Annexins contribute to biological processes such as endocytosis, exocytosis, cell division, apoptosis and growth regulation [24]; moreover, their role in the pathophysiology of inflammation, heart and circulation disorders, diabetes and cancer, has also been described [25]. More specifically, annexin A4 overexpression in solid tumors was widely reported [26, 27] and, more specifically, its contribution to chemoresistance (partly based on cellular efflux mediated by the copper transporter ATP7A) was clearly proved [28–30]. Our experiments led to a model of chemoresistance after paclitaxel treatment based on ANXA4 overexpression followed by its translocation to the inner side of plasma membrane; interestingly, Fhit overexpression avoided this subcellular relocalization thus restoring the sensitivity of cancer cells to paclitaxel-induced apoptosis, both *in vitro* and *in vivo* (Figure 1) [21].

We then narrowed the smallest region of the Fhit protein sequence still interacting with ANXA4 to a peptide ranging from position 7 to 13 of Fhit protein. This short sequence was not only able to bind ANXA4 but also to keep it in the cytosol during paclitaxel treatment, thus avoiding ANXA4 translocation to the inner side of cell membrane (Figure 1). Intriguingly, both *in vitro* and *in vivo* experiments on preclinical models of lung cancer demonstrated that Fhit peptide was effective in sensitizing lung cancer cells to paclitaxel treatment [31].

The resistance to the cytotoxic effects of anticancer drugs mainly represents the failure of pharmacologically-based therapies of cancer. Thus, overcoming chemoresistance, other than targeting mitogenic pathways, is one of the major goals in the development of novel anticancer drugs. A great example comes from the very recent FDA approval of the venetoclax, a drug able to target Bcl-2, an “unconventional” oncoprotein whose overexpression in cancer cells leads to resistance to apoptosis [32]. ANXA4 is another interesting “unconventional” oncoprotein to be targeted, being its overexpression involved in chemoresistance, although with different mechanisms compared to Bcl-2. An interesting opportunity comes from Fhit protein, a molecule lost early in the majority of human tumors, whose interaction with ANXA4 sensitize lung cancer cells to chemotherapy in a proof-of-principle preclinical setting of cancer treatment. Therefore, our long-lasting investigations not only pointed at Fhit as a molecular marker whose loss can negatively predict the outcome of cancer patients, but also as a candidate leading molecule for the development of therapeutic chemical compounds targeting ANXA4. The administration of conventional drugs plus Fhit-mimetic molecules in the treatment of patients bearing Fhit-negative tumors might be a fascinating perspective as it should increase their chemosensitivity thus maximizing the effects of chemotherapy itself. Of course, the path to reach an active small molecule mimicking Fhit activity in cancer cells is just at its very beginning; however, the need to generate anticancer drugs with novel mechanisms of action is urgent and clearly justify the pursuit of this innovative approach.
